# Reappraising the topology of cell-free fetal DNA in maternal blood toward improved prenatal genetic diagnostics

**DOI:** 10.1126/sciadv.aef5425

**Published:** 2026-10-02

**Authors:** Kieran Sparkes, Marnie Winter, Gustaaf A. Dekker, Lawrence Chamley, Martin J. Larsen, Benjamin Thierry

**Affiliations:** ^1^ Future Industries Institute, Adelaide University, Mawson Lakes, South Australia, Australia.; ^2^ Division of Women’s Health, Lyell McEwin Hospital and Robinson Institute, Adelaide University, Adelaide, South Australia, Australia.; ^3^ Department of Obstetrics and Gynaecology, University of Auckland, Auckland, New Zealand.; ^4^ Department of Clinical Genetics, Odense University Hospital, Odense, Denmark.

## Abstract

Noninvasive prenatal testing (NIPT) is a pregnancy screening test that analyses cell-free fetal DNA (cffDNA) in maternal plasma to detect fetal genetic abnormalities during pregnancy. Earlier and safer detection of fetal genetic abnormalities facilitated by NIPT has markedly transformed maternal-fetal medicine. However, NIPT is currently limited to be only a screening test due to fundamental biological bottlenecks, namely, the small proportion of cffDNA compared to cell-free maternal DNA in maternal plasma, the challenge to reliably distinguish between fetal and maternal DNA, and discordance between cffDNA and the true fetal genotype. Despite millions of NIPT being conducted each year, the exact origin and structural topology of cffDNA remain unexpectedly unclear. Elucidating the origin and structure of cffDNA could unlock new technological advances that address the current bottlenecks limiting NIPT toward realizing the full potential of state-of-the-art sequencing approaches for NIPT. In this Review, we critically discuss current cffDNA knowledge, including its origin, release into maternal blood circulation, and structures relevant to contemporary NIPT.

## INTRODUCTION

Noninvasive prenatal testing (NIPT) interrogates cell-free fetal DNA (cffDNA) circulating in maternal blood to provide genetic insights on the fetus from a 10-week gestation (*1*). Since its clinical introduction in 2011, tens of millions of NIPTs have been conducted worldwide, and its estimated market value exceeds US$8 billion (*1*, *2*). NIPT has transformed maternal-fetal medicine; in most cases, it provides reassurance about the health of an unborn child owing to the very high negative predictive values (>99%) for the three most common forms of trisomy, namely, Trisomy 21 Down’s, Trisomy 18 Edwards’, and Trisomy 13 Patau’s syndromes (*3*). This has led to a marked decrease in (now often avoidable) chorionic villus sampling and amniocentesis, following a negative NIPT result, although positive results still require confirmation by invasive diagnostic testing.

Despite the importance of NIPT in modern obstetrics, challenges persist in reducing the high false positivity and expanding diagnostic scope beyond being a screening test for large sized chromosomal abnormalities and selected microdeletions and microduplications. For instance, the positive predictive values across >73,000 tests were 96% for Trisomy 21 and 98% for Trisomy 18 but just 53% for Trisomy 13 (*4*). Furthermore, in a meta-analysis of >750,000 tests, 47.2% of all positive NIPT results were confirmed false (*5*).

The high false positivity and limited diagnostic scope of NIPT result from three interconnected fundamental biological bottlenecks. First, cffDNA constitutes only a small proportion of total cell-free DNA in maternal plasma compared to much more abundant cell-free maternal DNA. Second, distinguishing fetal from maternal DNA is challenging. Third, cffDNA does not always represent the true fetal genotype. This is because cffDNA is mainly derived from the placenta, which is normally genetically identical to the fetus except in cases of confined placental or fetal mosaicism, when a mutation arises independently in the placenta or fetus, respectively (*6*). Considering the predominant placental origin of cffDNA, the term cell-free placental DNA would better describe the origin of most cffDNA. However, as most studies to date do not distinguish between cell-free DNA of placental and fetal origin, cffDNA is used throughout this review to encompass the total circulating population of DNA from both origins, with placentally derived cffDNA specified where this origin has been explicitly demonstrated.

Advancing DNA sequencing technologies (Fig. 1) promise improved NIPT performance, but prohibitive costs currently preclude widespread integration and leave the fundamental biological bottlenecks unaddressed. Comprehensive fragmentomic and epigenetic studies (*7*) are now revealing the unique biological characteristics of cffDNA, with the potential to distinguish it from cell-free maternal DNA. Conversely, the structural topology of cffDNA is a core molecular characteristic, which remains unexpectedly poorly understood, with conflicting reports and no current consensus (*8*–*14*). Elucidating the structural topology (i.e., the structural organization and associations) of cffDNA could be used to distinguish it from cell-free maternal DNA. This knowledge could be leveraged to develop methods to specifically enrich cffDNA or deplete cell-free maternal DNA, for example, by harnessing differences in physical properties (such as size or density), molecular composition (such as extent of protein or lipid association), or surface markers (such as placental proteins), and as a result, increase the proportion of cffDNA compared to cell-free maternal DNA (fetal fraction). Toward addressing the challenge of confined fetal and placental mosaicism, it remains unclear whether placentally derived mosaic cffDNA has distinct molecular characteristics that could improve its detection. Regardless, enriching a purer population of cffDNA would enable more reliable identification of mosaicism (*15*). Bridging this knowledge gap would therefore likely translate into next-generation NIPT with expanded scope and even diagnostic potential without substantial added cost.

**Fig. 1.**
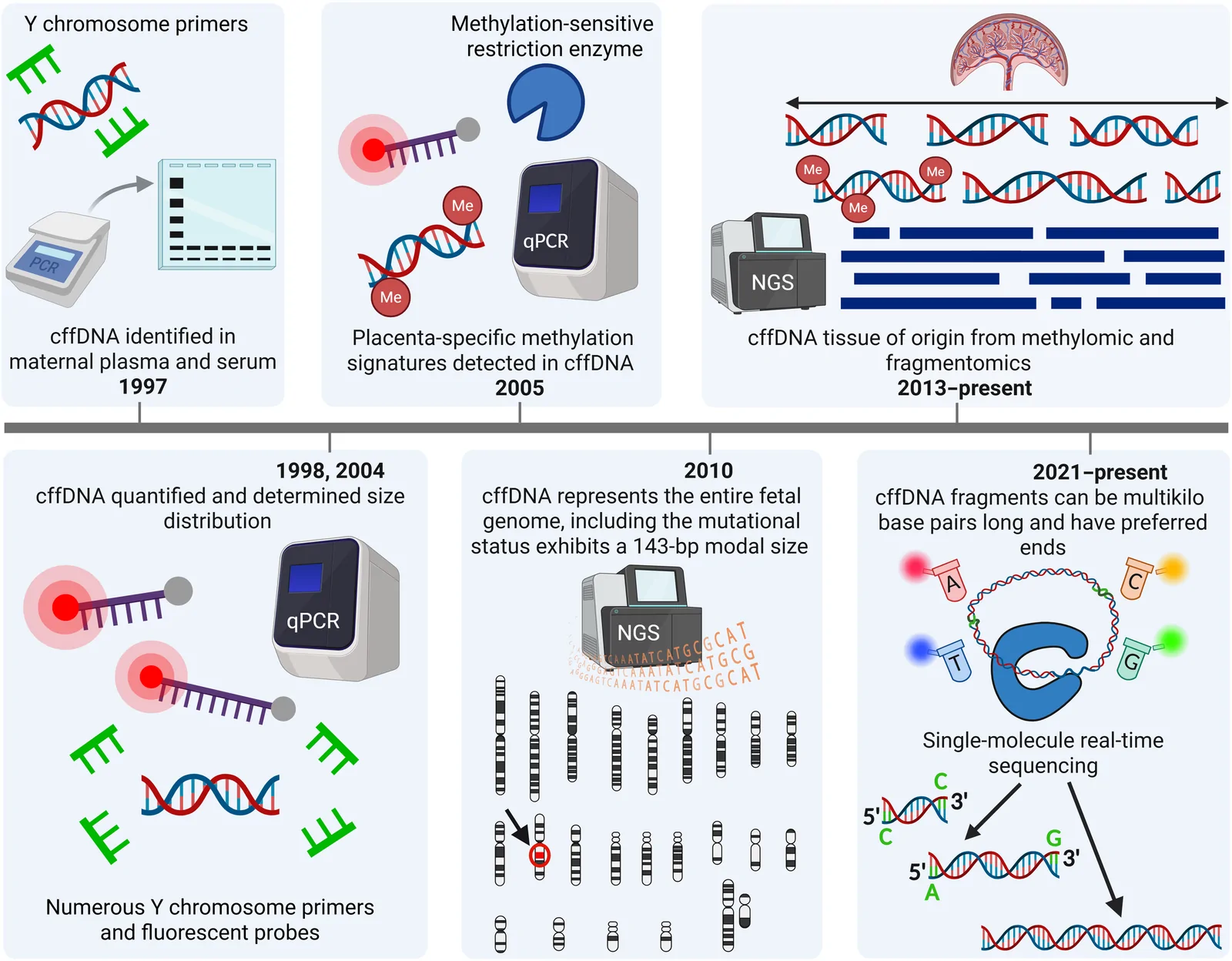
Advancing technologies in cell-free DNA research. Technological milestones in cffDNA research from discovery to advanced sequencing, revealing size profiles, methylation patterns, genomic content, tissue of origin, and fragment-end characteristics. qPCR, quantitative polymerase chain reaction. Created in BioRender. Sparkes, K. (2026); https://BioRender.com/4tw822b.

In this Review, we aim to critically reappraise the cellular and mechanistic origins of cffDNA to clarify its structural organization and associations with extracellular lipid- and protein-based structures in the maternal blood circulation. We discuss this current knowledge in the context of current NIPT and explore advances in noninvasive prenatal diagnostic that could result from an improved understanding of the structural topology of cffDNA.

## UNDERSTANDING THE ORIGIN OF CFFDNA

### Apoptotic origin of cffDNA

Since the discovery of cffDNA in maternal plasma in 1997, it has long been believed that cffDNA originates from apoptosis (*16*). Apoptosis is a highly regulated cell-death process characterized by distinct morphological changes and biochemical events, including DNA fragmentation and the release of cellular content in membrane-bound particles named apoptotic extracellular vesicles (EVs). Despite being a cell death mechanism, apoptosis is an essential process during placental development (*17*).

The exact proportion of cffDNA in maternal plasma, which is derived from apoptosis, is unknown but it is considered the primary origin because cffDNA has a characteristic 143–base pair (bp) modal fragment length, likely linked to a nuclease-cleaved nucleosome, alongside 10-bp periodicity, and preferential fragment ends also consistent with nuclease cleavage (*18*–*20*). However, other cell death mechanisms that generate some of these same features cannot be excluded as alternative origins of cffDNA (*7*).

A hallmark of apoptosis is the packaging of cellular contents, including fragmented DNA, into apoptotic EVs (often referred to as apoptotic bodies) and subsequent release into the extracellular space. Thus, since most cffDNA is believed to be apoptotically derived from the placenta, current understanding implicates placental apoptotic EVs in its release into maternal blood circulation, but to date there has been no mechanistic study directly investigating the exact underlying mechanisms.

While most cffDNA in maternal plasma exists as linear, mononucleosomal fragments ∼143 bp in length, cffDNA also exists as circular DNA (*21*), ultrashort ∼50-bp fragments (*22*), and longer >500-bp fragments (Fig. 1) (*23*). Unexpectedly, longer fragments were found to comprise 20.3% of cffDNA in the first trimester, with this proportion increasing with gestational age (*23*). Circular cffDNA is hypothesized to also originate from apoptosis due to its nucleosome-like structure and 10-bp periodicity (*21*). On the other hand, the unique fragmentation of ultrashort fragments suggests an alternative origin (*22*). The origin of long cffDNA fragments, which have been observed to be as large as 23,635 bp (*23*), could still involve apoptotic processes if the fragments are composed of multinucleosomal units. Conversely, it could indicate other origins such as necrosis, the second major cell death pathway, which releases long, irregular DNA fragments into blood circulation. Together, these findings demonstrate the heterogeneity of cffDNA fragments in maternal plasma and point to the fact that diverse mechanisms, distinct from those underpinning cell-free maternal DNA, likely govern its generation and processing, both of which remain poorly understood.

### Placental cellular origin of cffDNA

Compelling evidence for the placental origin of cffDNA arises from instances of confined placental mosaicism during pregnancy, when chromosomal aneuploidy, commonly a trisomy or monosomy X, identified in placentally derived cffDNA by NIPT was confined to the placenta and the fetus was unaffected (*24*). This is revealed by analyses of fetal genetic material obtained from invasive prenatal testing (e.g., amniocentesis) (*25*). In addition, methylation signatures of the placenta and cell-free DNA in maternal plasma, both at the single-gene (*26*, *27*) and across the methylome (*28*, *29*) have conclusively confirmed the placental origin of the majority of cffDNA.

Despite cffDNA primarily originating from the placenta rather than the fetus, it has been historically referred to as cffDNA on the basis that the placental genome is considered identical to that of the fetus, except in 2 to 3% of pregnancies with confined fetal and/or placental mosaicism (*6*). However, contrary to earlier belief that placental mosaicism is rare, recent evidence confirmed that the placenta undergoes early clonal evolution, creating genetic heterogeneity and allele variance from the fetus, as a typical feature of placental development (*30*, *31*). As a result, future studies should distinguish between cell-free DNA of fetal and placental origin, either through the use of separate terminology [cffDNA and cell-free placental DNA, respectively (*32*)] or by explicitly qualifying the origin when it is known because the genomic divergence between these origins directly affects the interpretation of NIPT.

### Trophoblastic origin of cffDNA in first-trimester pregnancy

At 10 weeks of gestation, when NIPT is typically conducted, the placenta is a discoid organ consisting of specialized cells organized into distinct structural units. Chorionic villi are branching fingerlike structures that facilitate exchange between the intervillous space of the placenta and maternal decidua (Fig. 2). The trophoblast cell lineage dominates placental structure, encompassing cytotrophoblasts, which continuously proliferate and fuse into the overlying syncytiotrophoblast in a process called syncytial fusion (*33*). A subset of cytotrophoblasts also differentiate into various subtypes of extravillous trophoblasts (Fig. 2), whose functions include anchoring the placenta and remodeling the maternal spiral arteries to ensure adequate fetal blood supply (*33*).

**Fig. 2.**
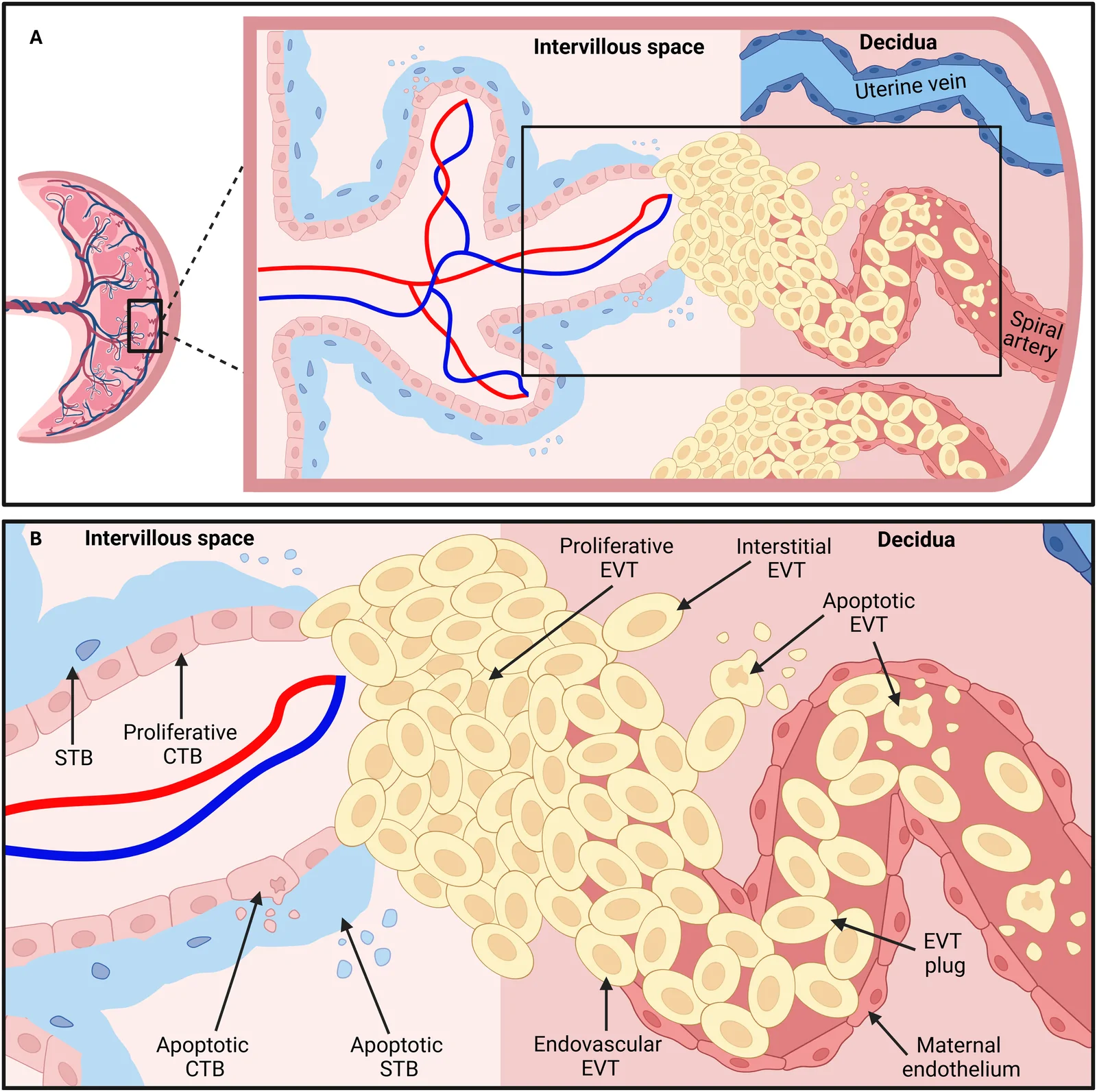
The different possible trophoblastic origins of cffDNA in first-trimester pregnancy. (**A**) Cross-sectional schematic of the human placenta at 7 to 10 weeks of gestation showing chorionic villi immersed in the maternal intervillous space, where maternal blood is delivered via spiral arteries and drained by uterine veins. Placental villi are lined by an underlying cytotrophoblast layer, which serves as the progenitor population for trophoblast renewal and the overlying syncytiotrophoblast, which facilitates nutrient exchange. (**B**) Magnified view of the intervillous space and decidua illustrating cytotrophoblast differentiation into the syncytiotrophoblast and extravillous trophoblasts. Proliferative extravillous trophoblasts invade maternal spiral arteries, forming a plug that modulates maternal blood flow, and differentiate into subtypes including interstitial extravillous trophoblasts, which anchor the placenta, and endovascular extravillous trophoblasts, which remodel spiral arteries. Apoptosis of the syncytiotrophoblast, extravillous trophoblasts, and cytotrophoblasts may contribute to the release of cffDNA into maternal blood circulation. CTB, cytotrophoblast; STB, syncytiotrophoblast; EVT, extravillous trophoblast. Created in BioRender. Sparkes, K. (2026); https://BioRender.com/jpj2lu8.

While the link between placental trophoblast cells and cffDNA is now well established, the exact contribution of different trophoblast subtypes in generating cffDNA remains unclear. The apoptotic origin of placentally derived cffDNA was early on hypothesized to be linked to the apoptotic-like process of syncytial fusion (*34*). The concentration of cffDNA in maternal plasma increases with gestational age (*35*), concordant with the rising turnover of cytotrophoblast cells fusing into the syncytiotrophoblast layer (*36*), providing correlational evidence for the role of syncytial fusion in generating cffDNA. However, it is unclear whether cytotrophoblasts themselves or the syncytiotrophoblast are the origin of cffDNA. First-trimester cytotrophoblasts exhibit more extensive apoptosis than the syncytiotrophoblast, are known to release cffDNA (*34*, *37*, *38*), and as the progenitor, they have been reported as the likely major origin of cffDNA (*39*). Conversely, supporting the syncytiotrophoblast origin hypothesis, ex vivo placental explant and cell studies showed that syncytiotrophoblast apoptosis is associated with DNA release (*40*) and that cffDNA is present in syncytiotrophoblast EVs (*41*, *42*).

Another possible origin is extravillous trophoblasts, which have substantial physiological roles in the first trimester of pregnancy and undergo a high rate of apoptosis, accompanied by shedding into maternal blood (*43*, *44*). A recent study combining single-cell transcriptomics with nucleosome spacing profiles identified extravillous trophoblasts as the primary source of cffDNA, followed by proliferative extravillous trophoblasts and villous cytotrophoblasts, with syncytiotrophoblasts contributing the least (*45*). This hierarchy was consistent in the first trimester and became more pronounced with gestational age. While this finding warrants further investigation, it underscores the unexpected knowledge gap on the cellular origin of cffDNA.

## UNDERSTANDING THE ORIGIN OF CELL-FREE MATERNAL DNA

Because cffDNA constitutes only a minor fraction of total cell-free DNA in maternal plasma, reliably distinguishing cell-free maternal DNA from cffDNA is essential to achieve sensitive and specific NIPT results. Cell-free maternal DNA is distinguishable from cffDNA by its longer modal fragment length of 166 bp, characteristic of a nucleosome (∼146 bp) and linker DNA (∼20 bp) generated from nuclease cleavage during apoptosis (*18*, *19*). The retention of linker DNA in cell-free maternal DNA is attributed to its higher level of methylation compared to cffDNA, which results in denser chromatin packing with histones and reduced susceptibility to cleavage (*18*).

Methylomic and fragmentomic studies in nonpregnant and pregnant individuals have conclusively confirmed the predominant hematopoietic origin of circulating adult cell-free DNA, primarily from leukocytes and monocytes (*19*, *45*–*48*). Chromatin immunoprecipitation sequencing (ChIP-seq) has also been adapted to investigate cell-free nucleosomes in plasma (*49*, *50*). Specifically, nucleosomes with histone modifications associated with active transcription were immunoprecipitated, and the associated DNA fragments were sequenced, revealing the chromatin signature, which was used to infer the cellular origin. In plasma from healthy nonpregnant individuals, ChIP-seq identified megakaryocytes (platelet progenitors) as a major origin of cell-free DNA, whereas erythroblasts (erythrocyte progenitors) contributed minimally (*49*). Notably, prior studies using cytosine-phosphate-guanine (CpG) methylation pattern analysis estimated that erythroblasts contribute 30% of cell-free DNA (*47*). This discrepancy likely arises from the challenge of distinguishing cell types with shared hematopoietic origins using methylation alone. Other minor tissue contributors include the liver, colon, heart, brain, and lung (*19*, *46*, *47*, *49*). Hence, cell-free maternal DNA has a more varied origin than cffDNA, and while the fragment characteristics still indicate a major apoptotic origin (*18*, *19*), there are probably various tissue-specific origins. For example, during a specialized form of programmed cell death named NETosis, neutrophils release neutrophil extracellular traps consisting of DNA, histones, and proteins, designed to trap and kill pathogens (*51*).

### Cell-free maternal DNA and NIPT

The unique physiological conditions of pregnancy also demand the consideration of alternative tissue-specific origins of cell-free maternal DNA. For instance, genome-wide methylation profiling of cell-free maternal DNA identified a stronger contribution of immune-cell derived cell-free DNA during pregnancy (*29*). Moreover, a recent large cohort study of singleton pregnancies undergoing cell-free DNA screening found that 7.7% had fibroids—benign uterine tumors, which contained genetic abnormalities in 50% of cases (*52*). Pregnancies with fibroids are approximately six times more likely to test false positive for subchromosomal aberrations (*52*). NIPT is also compromised by high maternal body mass index, which is associated with a reduced fetal fraction (*53*), due to alterations in the cell-free DNA profile potentially arising from inflammation or placental dysfunction. Considering the impact of cell-free maternal DNA on NIPT, further research is required to better understand its origin and structure, which is likely different to those of cffDNA, as this could pave the way to novel approaches to separate and enrich cffDNA from maternal plasma.

## UNDERSTANDING THE TOPOLOGY OF CFFDNA

Most cffDNA is believed to originate from apoptotic processes, which is commonly thought to implicate its extracellular release via apoptotic EVs (*41*). However, to date, no direct evidence supports this mechanism. In addition, once released into maternal blood circulation, EVs would likely interact with immune cells and extracellular enzymes or associate with other insoluble extracellular particles and cells (*54*). Extracellular particles is an umbrella term for all particles outside the cell. This includes EVs, which are lipid membrane–bound particles released by cells, and nonvesicular extracellular particles, which are multimolecular assemblies that lack a lipid bilayer (*55*). Hence, while the origin of cffDNA informs its initial structure (Fig. 3), directly interrogating its circulating structure is essential.

**Fig. 3.**
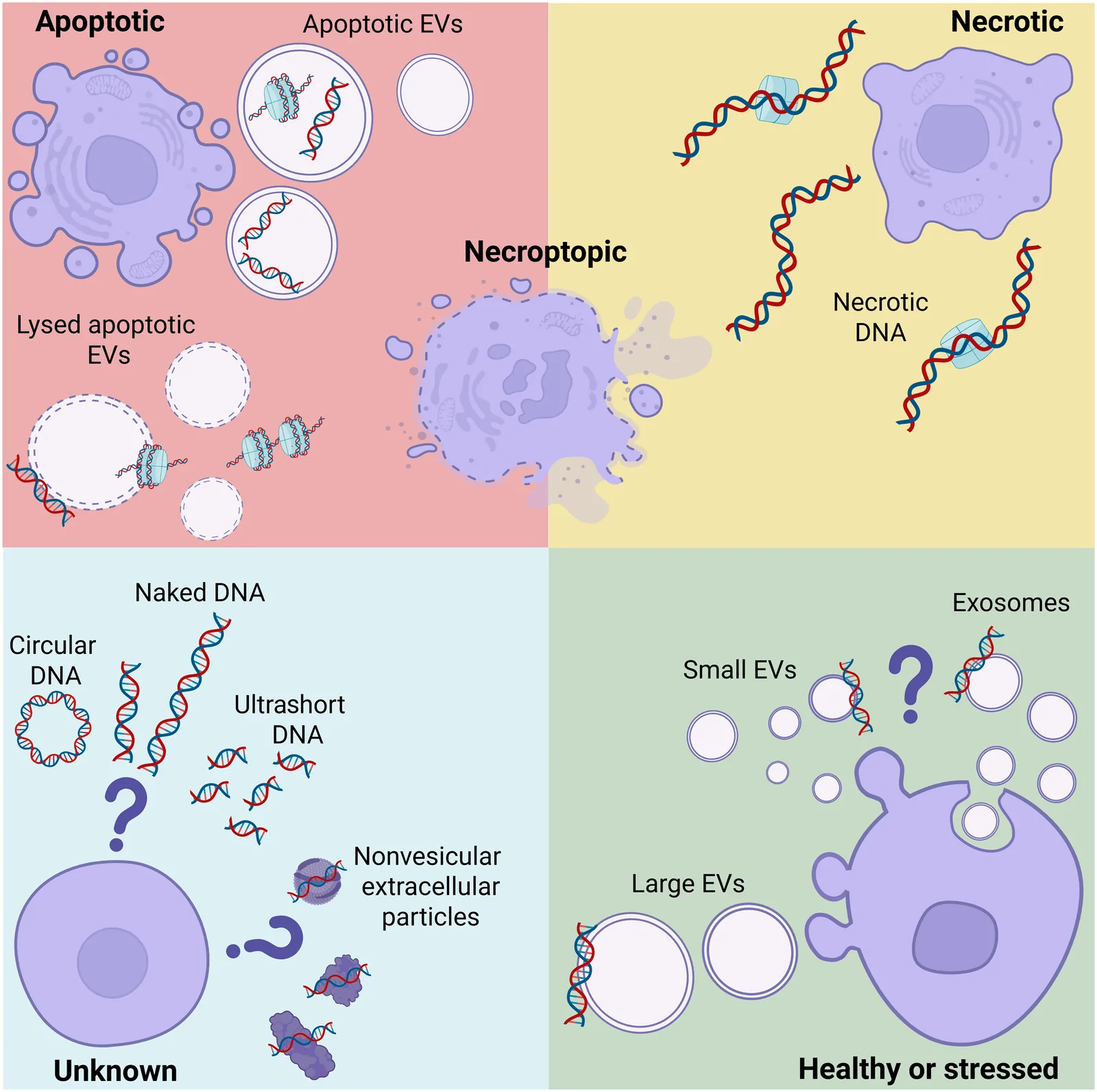
Possible structures of cffDNA. Apoptotic cells may release cffDNA in apoptotic EVs, which may become lysed to release their contents. Necrotic cells could generate long, irregular fragments of cffDNA. Necroptotic cells may release cffDNA with structural characteristics of apoptotic and/or necrotic cells. Alternatively, healthy and stressed cells may release cffDNA in large EVs via outward budding or in small EVs either by outward budding or as exosomes formed through endosomal maturation. Naked fetal DNA, including circular DNA and ultrashort DNA, alongside nonvesicular extracellular particles (e.g., DNA-protein-lipid complexes, termed virtosomes) associated with cffDNA could be released by cells under various physiological conditions, which are currently unknown. Created in BioRender. Sparkes, K. (2026); https://BioRender.com/b8fyd2b.

### cffDNA association with EVs

Circulating EVs are extensively heterogeneous in size, biogenesis, and cellular origin, so studies typically focus on the association of cffDNA with a subset of EVs. Given the major placental and apoptotic origin of cffDNA, it was initially thought that large (>200 nm in diameter) apoptotic placental EVs were associated with a substantial proportion of cffDNA (*8*, *41*), yet only limited experimental evidence supports this hypothesis. Orozco *et al.* (*56*) enriched large EVs by low-speed centrifugation from both a human trophoblastic cell line and plasma from pregnancies (*9*), and using flow cytometry observed an association with DNA, including for large EVs bearing either the syncytiotrophoblast marker placental alkaline phosphatase or the extravillous trophoblast marker, human leukocyte antigen–G. However, the lack of rigorous characterization of the enriched EV fraction and basic flow cytometry methodology means that contamination with cellular debris cannot be excluded. Further studies are warranted to confirm the association between large EVs and fetal and placental DNA and quantify it.

Small (<200 nm in diameter) EVs have garnered substantially more attention due to their demonstrated roles under multiple pathophysiological conditions. A specific focus has been on exosomes, a small EV subtype, which are actively secreted by cells and are defined by their biogenesis through a multivesicular body dependent mechanism. Correlational evidence for the association of cffDNA with small EVs includes their increasing concentration in the plasma during pregnancy (*57*), which coincides with the increasing concentration of cffDNA with gestational age (*35*). Multiple studies have processed plasma and serum from maternal blood by ultracentrifugation or with polymer-precipitation kits to enrich small EVs and detected cffDNA in the EV fraction (summarized in table S1) (*10*–*13*), suggesting a structural association between cffDNA and small EVs. To our knowledge, only one study (*10*) has investigated the proportion of cffDNA in the EV-enriched fraction versus in total plasma, reported at 8.7%, while the reported proportion of total cell-free DNA in maternal plasma present in the EV-enriched fraction varies hugely (from 10.3 to 52.9%) (*10*, *12*).

The commonly used physical methods (e.g., ultracentrifugation) of EV enrichment mean that these studies were probably analyzing a heterogeneous population of extracellular particles (*55*), including nonvesicular extracellular particles such as DNA-protein complexes (e.g., nucleosomes) and DNA-protein-lipid complexes (virtosomes). These multimolecular assemblies are released into the extracellular space through various mechanisms and differ to EVs according to the absence of a lipid membrane. Hence, reports on the association of small EVs and exosomes with cffDNA should be interpreted with caution, and to date, it remains unclear whether cffDNA is associated with EVs or with nonvesicular extracellular particles. It is also unknown whether these extracellular particles are exclusively derived from the placenta, what proportion of the total plasma cffDNA this structural association represents, and whether it represents a subpopulation of cffDNA with distinct molecular fragmentation.

## TOPOLOGY OF EXTRACELLULAR PARTICLE DNA: LESSONS BEYOND PREGNANCY

### Healthy and circulating tumor DNA association with EVs

The presence of DNA in small EVs was first reported following enrichment of EVs by ultracentrifugation of healthy human plasma in 2013 (*58*). Circulating tumor DNA was subsequently detected in EVs enriched by ultracentrifugation of serum from patients with pancreatic cancer in 2014 (*59*). Since then, numerous studies have reported an association of both healthy and circulating tumor DNA with EVs (*60*). While quantitative data are limited, EV-associated DNA (EV-DNA) typically accounts for only a small proportion of the total plasma DNA in healthy donors (*61*, *62*) and patients with cancer (*63*–*65*). On the other hand, Fernando *et al.* (*66*) reported that 90% of total cell-free DNA in plasma from healthy donors was in the small EV fraction, whereas Vagner *et al.* (*67*) reported that large EVs carry most of the circulating tumor DNA in the plasma of patients with prostate cancer. A handful of studies have proposed mechanisms for the loading of nuclear DNA into exosomes (*68*, *69*), but there is currently no consensus.

As in the case for cffDNA, varying degrees of analytical rigor have been applied to characterize enriched EV fractions in the broader cell-free DNA field and the extent of contamination with nonvesicular extracellular particles, which likely greatly contributes to the lack of consensus in the field. For example, the seminal study by Jeppesen *et al.* (*70*) reported that following extensive enrichment by density-gradient ultracentrifugation and direct immunoaffinity capture, alongside rigorous characterization, the population of classical exosomes and small EVs enriched from five cancer cell lines and primary human kidney epithelial cells did not contain double-stranded genomic DNA or histones. The authors hypothesized that previous reports of DNA associated with small EVs were likely artifacts resulting from impure preparations that included nonvesicular contaminants, highlighting the importance of the enrichment methodology and precise phenotyping. A DNA-targeted immunoprecipitation approach further questioned the prevalent EV-DNA association dogma, providing compelling evidence in both cell line cultures and plasma of patients with cancer that nuclear cell-free DNA is not associated with EVs but rather is predominantly associated with nucleosomal particles (*64*).

### Nonvesicular extracellular particles

Nonvesicular extracellular particles are frequently cited as a being associated with all types of cell-free DNA (i.e., “healthy,” fetal and placental, and tumor), based on the presence of cell-free DNA in EV-depleted or EV-free plasma fractions (e.g., following size exclusion chromatography) (*71*), and the protection these structures may be expected to provide against cell-free DNA degradation in the peripheral blood circulation. However, while a handful of studies have proposed mechanisms for the release of nonvesicular DNA (*64*, *70*), specific enrichment has yielded inconsistent findings. Nonetheless, the recent adaptation of ChIP-seq to immunoprecipitate and sequence DNA-histone structures in the plasma of healthy donors and patients with metastatic colorectal carcinoma demonstrates that at least a fraction of both cell-free healthy and tumor DNA in plasma is bound to histones in a nucleosome structure (*49*).

Similar to cell-free healthy and tumor DNA, the molecular features of cffDNA also implicate its release and circulation in maternal plasma as nucleosomes (*18*, *50*). The nucleosome association hypothesis could also be compatible with the postpartum degradation of cffDNA, which occurs in a rapid initial stage in the first 2 hours, followed by a slower clearance phase with a half-life of 13 hours (*72*). However, to our knowledge, there is to date no peer-reviewed study that has specifically enriched cffDNA associated with nonvesicular extracellular particles. Hence, it is unclear what release mechanism could be involved, the proportion of cffDNA that is associated, or the structural form it could assume if released, such as being incorporated into larger macromolecular assemblies.

### Methodological challenges regarding extracellular particle classification and enrichment

Blood is a complex biofluid containing numerous types of extracellular particles, including several EV subtypes and a broad range of nonvesicular extracellular particles, with often overlapping physical properties including size and density, making selective isolation particularly challenging (Fig. 4) (*55*). As a result, we use the term enrichment rather than isolation in most cases to reflect the fact that efficient separation of specific extracellular particle subtypes is extremely difficult using physicochemical methodologies.

**Fig. 4.**
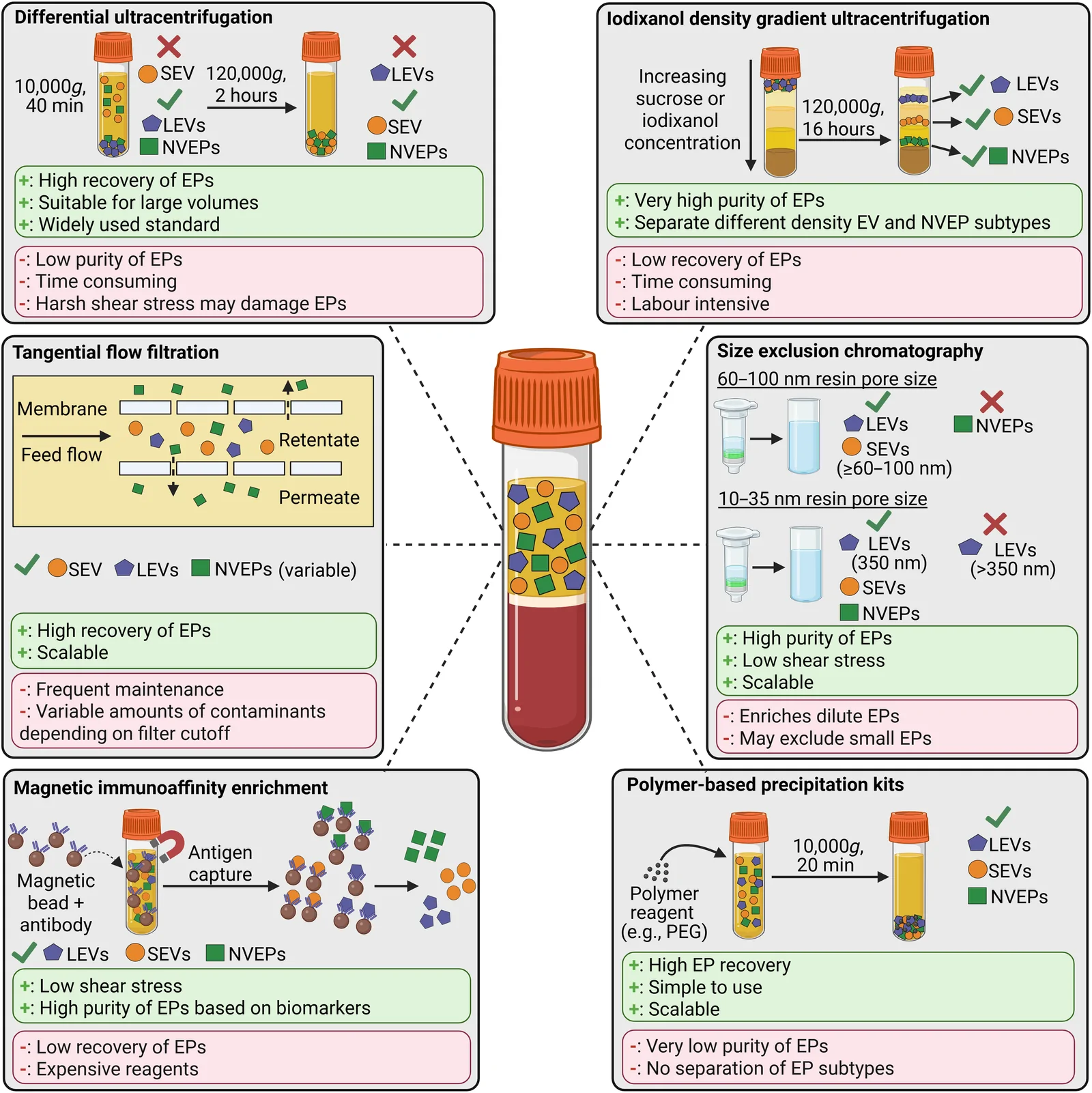
Comparison of extracellular particle enrichment techniques. Findings on the association of cffDNA with EVs and nonvesicular extracellular particles are affected by the blood processing and enrichment technique used. Each process and technique separates different extracellular particles, which may be associated with cffDNA. EP, extracellular particle; LEV, large EV; SEV, small EV; NVEP, nonvesicular extracellular particle; PEG, polyethylene glycol. Created in BioRender. Sparkes, K. (2026); https://BioRender.com/b4o0o3q.

These methodological constraints have likely contributed to the misattribution of cell-free DNA to certain extracellular particles. A case in point is exomeres (∼35-nm diameter), a nonvesicular extracellular particle subtype that lacks classical exosome markers (e.g., CD9, CD63, and CD81) but does carry RNA, DNA, and proteins previously attributed to small EVs/exosomes in nonpregnant individuals (*73*). Supermeres (∼27-nm diameter) represent another recently found nonvesicular extracellular particle subtype with cargo profiles distinct from small EVs/exosomes. While to date, supermeres have only been shown to carry RNA and protein (*74*) and not DNA, their discovery underscores the growing recognition of existence of diverse nanosized extracellular particle populations. Further studies are warranted to settle the debate surrounding the association of cell-free DNA with EVs and nonvesicular extracellular particles.

Another well-recognized challenge is the use of imprecise or outdated extracellular particle classification and a lack of rigorous phenotyping in many studies. Exosomes are a good example because although they are defined by their biogenesis, exosome studies most frequently rely on physical methods for enrichment, which has contributed to conflicting reports about whether exosomes carry DNA (*60*). Implementation of rigorous preanalytical reporting following the Minimal Information for Studies of Extracellular Vesicles guidelines (*55*) is therefore critical to ensure traceability, standardization, and accurate interpretation of results.

## ALTERNATIVE ORIGINS OF CIRCULATING FETAL DNA

### Syncytial nuclear aggregates

Syncytial nuclear aggregates are clusters of >10 membrane enclosed, apoptotically derived nuclei released by the syncytiotrophoblast into the blood from as early as 6 weeks of gestation (*75*). Although an estimated 100,000 syncytial nuclear aggregates are released per day into maternal blood in healthy pregnancies (*76*), their concentration in the maternal peripheral blood circulation is very low (*77*). The rarity of syncytial nuclear aggregates is most likely due to their large size (20- to 210-μm diameter) that prevents circulation beyond the pulmonary capillaries where they are degraded and cleared (*78*). Syncytial nuclear aggregates do exhibit signs of apoptosis including the presence of activated caspase-3, cleaved cytokeratin, and terminal deoxynucleotidyl transferase–mediated deoxyuridine triphosphate nick end labeling–positive (TUNEL) staining (*76*), and considering the large amount of DNA that is harbored in >10 syncytiotrophoblast nuclei, one cannot exclude the possibility that syncytial nuclear aggregates contribute a meaningful amount of placentally derived cffDNA. Syncytial nuclear aggregates have EVs budding from the surface (*76*), and while it remains to be proven if these EVs harbor DNA, it could be another plausible mechanism contributing placentally derived cffDNA into maternal blood circulation.

### Amniotic fluid

Amniotic fluid is created from maternal plasma in the first trimester, but later in pregnancy, it largely consists of fetal urine (*79*). It forms a protective layer that surrounds the fetus during pregnancy. Prenatal genetic diagnosis by amniocentesis traditionally involves the analysis of fetal cells present in the fluid (from 15 weeks of gestation), most commonly by karyotyping or chromosomal microarray analysis. In contrast, the cell-free fraction of amniotic fluid has received comparatively little attention, despite containing ∼200-fold more fetal DNA per milliliter than maternal plasma (*80*). This raises the possibility that amniotic fluid may act as a secondary origin of cffDNA, with DNA potentially transferring across the placenta into maternal blood circulation via a concentration gradient (*80*). However, amniotic fluid DNA is unlikely to contribute substantially to circulating cffDNA, as it is largely maternal in origin during the first trimester, is more fragmented, and lacks the placenta-specific DNA methylation signatures characteristic of cffDNA (*81*, *82*).

### Circulating trophoblasts

The bidirectional transfer of cells between fetus and placenta and mother, known as fetomaternal microchimerism, is well established. A substantial proportion of these cells undergo apoptosis (*83*), potentially generating placentally derived cffDNA. Among circulating fetal and placental cells, nucleated fetal red blood cells and trophoblasts are the most common with fetal leukocytes and fetal progenitor/stem cells also being identified but at very low frequencies (*84*). As discussed above, conclusive evidence supports a major trophoblastic origin for cffDNA (*25*–*28*, *39*), effectively excluding fetal nucleated red blood cells, lymphocytes, and progenitor/stem cells as a major source.

Estimates of circulating trophoblast concentration in maternal plasma vary considerably due to enrichment challenges. Higher estimates (2 to 6 cells/ml) (*85*) suggest that they could account for 10 to 29% of the ∼21 fetal genome equivalents/ml of maternal plasma in the first trimester (*86*), whereas more conservative estimates (0.2 cells/ml) (*87*) imply a possible contribution of only ∼1%. It remains unclear whether the scarcity of circulating trophoblasts reflects limited extrusion, frequent entrapment in pulmonary capillaries, and/or rapid clearance and degradation, all of which have implications for their contribution to cffDNA.

The idea of a cell-based NIPT using fetal lymphocytes was first proposed in 1969 (*88*), but the long-term survival of fetal leukocytes and progenitor/stem cells in maternal blood circulation postpartum (*89*, *90*) effectively excludes their use due to the risk of contamination from previous pregnancies. In contrast, circulating nucleated fetal red blood cells and trophoblasts are more viable candidates for cell-based NIPT, given their rapid clearance following delivery (*91*, *92*). However, the rarity, lack of specific markers, and often extensive apoptosis have posed major challenges to widespread clinical adoption of cell-based NIPT (*93*). Nevertheless, enrichment strategies of circulating trophoblasts and nucleated fetal red blood cells have enabled single-cell next-generation sequencing (NGS) for the detection of fetal genetic disorders (*87*, *94*–*96*). Furthermore, ARCEDI has developed the EVITA TEST COMPLETE, which is the first commercially available cell-based NIPT that isolates circulating trophoblast to detect chromosomal aberrations, microdeletions (≥2 mega–base pairs), and microduplications (≥5 Mb), with the potential to screen for mutations across the entire fetal genome (*97*). Accordingly, the potential for a cell-based NIPT to capture intact diploid fetal genomes and for a nucleated fetal cell–based NIPT to circumvent false results arising from confined fetal and placental mosaicism could provide advantages over current cffDNA-based NIPT (*98*), which primarily analyzes placentally derived cffDNA and warrants further research.

## CURRENT AND EMERGING NIPT

### Clinically standard NIPT

Clinically standard NIPT currently uses low-depth (0.1 to 0.5×) genome-wide NGS. Chromosomal aberrations, including Trisomy 21, Trisomy 18, and Trisomy 13, as well as, in some cases, numerical sex chromosome aberrations, rare autosomal trisomies, copy number variations, and microdeletions and microduplications, are detected using a count-based approach and assigned to the fetus under the presumption that the conditions are absent in the mother. NIPT is typically conducted at 10 weeks of gestation when the median fetal fraction is relatively low (10 to 15%) (*35*), representing a practical compromise between cost, adequate test performance, and early detection of abnormalities, to enable timely pregnancy management and informed decision-making.

Beyond the three most common trisomies, NIPT positive predictive values are significantly lower, with the false-positive rate reaching 84.5% for rare autosomal trisomies (*5*), representing a well-documented ethical concern (*99*, *100*). This variation is largely attributable to the fact that positive predictive value is prevalence dependent. As chromosomal conditions occur in only 0.002 to 0.4% of pregnancies (*4*, *5*), each false-positive result exerts a disproportionate effect on the false-positive rate and positive predictive value for rarer conditions. Chromosome-specific factors contribute additionally because larger chromosomes yield a higher dosage of cell-free DNA and therefore greater counting precision during sequencing analysis, whereas discrimination of the sex chromosomes is complicated by the high homology between X and Y (*101*). Moreover, clinically accessible NIPT is typically limited to screening for chromosomal aneuploidies and only some microdeletions and microduplications, omitting monogenic disorders such as spinal muscular atrophy, Tay-Sachs disease, and Achondroplasia, which are responsible for severe paediatric complications, early childhood deaths, and long-term morbidities (*102*). Although detecting fetal monogenic disorders based on a maternal blood sample is technically feasible, the additional cost and complexity have limited widespread clinical implementation.

Expanded NIPT is desirable as it would enable early detection of thousands of severe genetic disorders, which would otherwise go undetected until birth or even years later. This would enable parents to make informed reproductive choices, including termination or preparation for specialized care, and would also guide reproductive planning for future pregnancies (e.g., carrier screening and preimplantation genetic testing). Furthermore, in some cases, early detection provides the opportunity for early treatment that prevents or substantially reduces pathophysiology.

### Emerging NIPT technologies

Emerging NGS approaches such as deep targeted genome sequencing, deep whole/targeted exome sequencing, and deep trio-exome sequencing, which uses reference samples from both parents, are capable of expanding NIPT to detect small-sized mutations, including single nucleotide variants. Cell-free DNA from maternal plasma subjected to deep exome- or trio-exome sequencing enables accurate detection of fetal chromosomal aberrations, larger (3 to 20 Mb) copy number variants, insertions and deletions, and single nucleotide variants, with 100% concordance to invasively obtained fetal samples (*103*, *104*). Furthermore, in a recent large cohort study, deep targeted genome and exome sequencing enabled a comprehensive cffDNA screen, which detected fetal aneuploidies, microdeletions, and single gene variants and collectively provided a 60.7% increased detection rate for diagnostic genetic variants compared to current standard screening (*105*).

NGS uses pair-end reads and is inherently biased toward shorter (<500 bp) DNA templates (*23*). Because the modal length of cffDNA in maternal plasma is 143 bp and most fragments are <200 bp (*18*, *106*), for NIPT, this limitation of NGS has often been overlooked, disregarding the potential significance of larger multikilobase cffDNA fragments in plasma, which are actually more abundant than initially thought (*23*). Long cffDNA fragments may contain multiple mutations and/or CpG methylated sites, potentially paving the way for additional genetic and epigenetic insights from NIPT. The diagnostic benefit of analyzing long fragments for NIPT remains to be established, but the capability of third-generation sequencing technologies to analyze all lengths of DNA is a promising prospect (*107*, *108*).

### Predicting pregnancy complications

Given that cffDNA primarily originates from placental cell turnover (i.e., apoptosis), its analysis has the potential to offer insights into placental health and identify placental stress, damage, or dysfunction to detect or predict pregnancy complications. For instance, when NIPT detects a chromosomal aneuploidy that is confirmed as a case of confined placental mosaicism by a nonmosaic amniocentesis, the fraction of placentally derived cffDNA with a chromosomal aneuploidy relative to nonmosaic cffDNA (mosaic ratio) correlates with increased risk of adverse pregnancy outcome (*109*), highlighting the feasibility of using NIPT as a first-tier screening tool for predicting pregnancy complications.

Preeclampsia arises from placental dysfunction, with early-onset preeclampsia (before 34 weeks of gestation) characterized by incomplete spiral artery remodeling, leading to insufficient blood supply to the fetus, while late-onset preeclampsia (after 34 weeks of gestation) typically involves normal placentation and is driven by maternal systemic dysfunction later in pregnancy (*110*). Both early- and late-onset preeclampsias result in the often-rapid onset of hypertension, proteinuria, and edema, which have severe health implications for both mother and fetus (*110*). Early diagnosis and treatment substantially improve outcomes (*111*), yet there is currently no gold standard for predicting preeclampsia before symptoms manifest and current models based on maternal characteristics (e.g., body mass index and blood pressure) and blood protein biomarkers suffer from a low positive predictive value (*112*). It has long been known that preeclampsia is associated with widespread apoptosis of placental trophoblasts (*113*), leading to the logical presumption that placentally derived cffDNA release is altered. Evidence suggests this is the case, with total cell-free DNA concentration in maternal plasma often elevated in affected pregnancies and a low fetal fraction associated with increased risk of complications (*114*). Together, these parameters can be assessed alongside routinely available maternal characteristics to improve prediction of preeclampsia (*114*).

Additional analysis of NGS data collected for NIPT, also aimed at identifying cell-free DNA profiles unique to preeclampsia, includes nucleosome profiling, which can quantify maternal and fetal tissue signatures to reveal early placental and endothelial dysfunction to predict preeclampsia (*115*) and other common complications such as fetal growth restriction (*116*) and preterm birth (*117*). Alternatively, modified DNA purification, library preparation, and sequencing can identify molecular characteristics of cffDNA specific to pregnancy complications for use as predictive markers. For example, the genome coverage of ultrashort cell-free DNA fragments (∼50 bp) in maternal plasma is different in individuals with preeclampsia compared to healthy pregnancies (*22*). Distinct cell-free DNA methylation signatures have also been observed in preeclampsia and, when combined with maternal characteristic screening, improve the sensitivity of early-onset preeclampsia prediction (*118*). In parallel, third-generation sequencing such as the Oxford Nanopore sequencing and PacBio’s single-molecule real-time sequencing platforms, which can directly detect methylation and fragmentation signatures (*23*, *119*), offers the potential for low-cost, decentralized, point-of-care screening for pregnancy complications.

## FUTURE PERSPECTIVES FOR NIPT

### Current challenges for NIPT

The relatively low proportion of cffDNA compared to cell-free maternal DNA in maternal plasma and the absence of a “perfect” molecular biomarker, such as fragment size or CpG methylation status, to discriminate between maternal and fetal genetic aberrations represent fundamental biological challenges contributing to the problematic false-positive rate (*5*), test failures (*4*), and more generally the limited testing scope of NIPT. These challenges are further exacerbated when there is a low fetal fraction due to maternal factors, such as high body mass index, or by comorbidities such as maternal cancer (*35*, *120*). False positive NIPT results are often attributed to cases of confined fetal and placental mosaicism or vanishing twin syndrome, which only occurs in 2 to 3 and <1% of all pregnancies, respectively (*6*, *121*). While these conditions may be overrepresented in false-positive cases, we believe that the reduced number of fetal NGS reads and misclassification of reads due to the high cell-free maternal DNA background, which on average exceeds cffDNA 10:1, should not be underestimated as a contributor to both false-negative and false-positive NIPT results.

Emerging deep trio- and individual-targeted genome and targeted/whole exome sequencing approaches promise highly refined separation of cffDNA from cell-free maternal DNA, capable of detecting single nucleotide variants, but the extreme depth of NGS necessitated by the relative rarity of cffDNA is a bottleneck to the widespread clinical integration of these technologies. Artificial intelligent–driven algorithms capable of optimizing bioinformatic protocols (*122*) and reducing NGS read depth, coupled with declining DNA sequencing costs, promise to enhance efficiency and affordability of these approaches. However, simpler separation of cffDNA from cell-free maternal DNA and increasing the fetal fraction remain the most straightforward strategies toward improving NIPT and leveraging these advancements.

### Increasing the fetal fraction

Cell-free DNA used for NIPT is typically extracted from maternal plasma using silica-based column techniques, which extract the total cell-free DNA (i.e., maternal and fetal DNA). Fetal fraction enrichment based on size selection of smaller fetal fragments (<160 bp) has shown promise, increasing the fetal fraction approximately two- to threefold (*123*–*126*). Enrichment reduces maternal background and, in pilot studies, lowered NIPT failure rate (*125*) and improved the positive predictive value for the detection of the three most common trisomies (Trisomy 13, 18, and 21) from 59.3 to 95.9% (*127*). Nonetheless, fetal fraction enrichment by fragment size selection is inherently limited because of the heterogeneous lengths of cell-free DNA, which means a large proportion of shorter cell-free maternal DNA remains included for analysis and longer cffDNA is excluded. Furthermore, fragment size–based fetal DNA enrichment is always a trade-off between increasing the fetal fraction while retaining enough total DNA material for sequencing, which is of heightened importance for reliable deep sequencing. Hence, it stands to reason that an enhanced understanding of cffDNA’s structure and likely associations with extracellular particles could be leveraged to allow more efficient enrichment of cffDNA and could be pivotal for unlocking NIPT performance.

### Fetal DNA enrichment: Lessons from liquid biopsies in oncology

Circulating tumor DNA is one of the most promising advances in oncology, with the potential to vastly improve how cancer is detected and treated. Conversely, EVs and particularly exosomes are increasingly recognized as key mediators of intercellular communication in cancer, contributing to tumor progression and metastasis (*128*). This functional relevance is reflected by their increased presence in cancer (*128*) and has positioned them as promising sources of tumor nucleic acid materials for liquid biopsy. Several studies have reported that liquid biopsy based on DNA in extracellular particles may improve sensitivity and specificity, particularly in early stages of disease and residual disease where only a limited amount of circulating tumor DNA is present in the peripheral circulation (*129*–*132*). Specific enrichment of tumor EVs has shown promise in reducing false results from background healthy cell-free DNA and also warrants further investigation (*133*, *134*). In addition, the recent adaptation of ChIP-seq to investigate plasma nucleosomes associated with actively transcribed genes can delineate tumor gene expression levels (*49*, *135*–*138*) with the potential to assess disease progression and monitor treatment response. DNA found in extracellular particles enriched via physical methodology from the plasma of a patient with cancer has been found to be enriched in long, multikilobase DNA fragments (*59*, *63*, *67*), which may provide greater diagnostic power over highly fragmented plasma cell–free DNA. Hence, considering the unquestionable benefits of increasing the fetal fraction in NIPT and anticipated diagnostic advantage of testing large DNA fragments, lessons from cancer liquid biopsies suggest that targeted enrichment of placentally derived extracellular particles with known association to fetal DNA may represent a useful strategy that is yet to be tested.

### Expanded NIPT toward in utero and perinatal therapeutics

Increasing the fetal fraction could expand the scope of NIPT while maintaining affordability by reducing the need for deep sequencing or unlocking NIPT with alternative technologies (*139*), enabling early detection of a broader range of genetic conditions. Some of these conditions can be treated during the prenatal developmental window to reduce, stop, or prevent disease pathophysiology. For example, in utero enzyme replacement therapy is one approach for treating metabolic disorders. In a recent landmark study (*140*), a fetus with infantile-onset Pompe disease, a lysosomal storage disorder usually fatal by age 2, received enzyme replacement therapy from 24 + 5 weeks of gestation, followed by standard postnatal care. At 13 months of age (time of publication), the child showed normal biomarker levels, cardiac and motor function, and age-appropriate developmental milestones. Alternatively, certain monogenic disorders resulting from protein dysfunction can be treated in utero with small-molecule drugs. In one case (*141*), a mother carrying a fetus at risk for type 1 (severe) spinal muscular atrophy received Risdiplam orally from 32 + 5 weeks of gestation. Combined with postnatal therapy, the child showed no features of spinal muscular atrophy up to 30 months of age (time of publication).

Moreover, since the advent of CRISPR-based technologies in 2012, recent years have seen meaningful advances in vector engineering and gene editing with the more precise base and prime editing (*142*). Multiple preclinical murine studies demonstrate the feasibility of in utero gene therapy (*143*–*145*), but to date, there are no in utero in-human trials. In utero gene therapy carries particularly high stakes, as off-target effects are more challenging to monitor and may have lifelong consequences. Combined with the risks of invasive delivery procedures, this has driven demand for improved delivery systems, including viral vectors with reduced immunogenicity and nonviral vectors, such as polymer and lipid nanoparticles, with optimized formulations and targeting (*146*) to achieve greater stability, transfection efficiency, and sustained expression.

mRNA–lipid nanoparticle platforms (consisting of mRNA encoding the editor enzyme, the lipid nanoparticle, and customized guide RNA) have achieved targeted editing of fetal haematopoietic stem cells (*147*), the brain (*148*, *149*), and multiple organs (*150*) in murine models. Toward clinical application, the “United States Platform Technologies Provision” enacted in 2022 has presented a clear opportunity for the application of mRNA–lipid nanoparticle platforms to address *N*-of-1 disorders (*151*). In a major milestone, personalized lipid nanoparticle base editing has been used to correct a mutation associated with carbamoyl-phosphate-synthetase-1 deficiency in a baby treated at 7 and 8 months of age. In the 7 weeks postinfusion, the patient had improved metabolic control (*152*). While further follow up is required, one major advantage of lipid nanoparticle vectors is the potential for readministration, unlike adeno-associated viral vectors, which are limited by their immunogenicity (*153*).

Given the additional challenges (e.g., maternal and fetal safety, invasive delivery, and off-target effects) of in utero human gene therapy, routine clinical application remains uncertain. Nonetheless, its rapid customizability and deployment combined with the potential for early detection of conditions through expanded NIPT make it particularly promising for improving outcomes in cases of severe neonatal genetic disorders.

## ETHICAL, LEGAL, AND REGULATORY CONSIDERATIONS FOR NIPT

As NIPT looks to expand toward potential broader condition detection, diagnostic results, and integration with in utero therapeutics, it is important to consider its increasingly complex ethical, legal, and regulatory landscape (*154*). Pre- and posttest genetic counseling should be a core, nonnegotiable component of NIPT. Information must be accurate, balanced, and nondirective to ensure that decisions arise with full understanding of their implications. This is particularly important given the risk of false-positive results, which cause considerable psychological distress during the weeks preceding invasive diagnostic confirmation or, in some cases, prompt a misinformed decision to terminate an otherwise healthy pregnancy (*155*). Furthermore, counseling is essential for sensitive incidental findings such as maternal malignancies that carry major psychological and clinical consequences for which patients may be wholly unprepared (*100*).

Genetic counseling also carries direct medicolegal weight, and negligence remains the principal basis for litigation in prenatal testing, often arising from inadequate disclosure of the fact that NIPT is a screening rather than diagnostic tool, failure to discuss confirmatory invasive testing, or misinterpretation of laboratory results (*156*). These failures may give rise to claims of wrongful birth, wrongful life, or wrongful abortion, which carry substantial legal and ethical complexity across jurisdictions (*154*). Professional guidelines therefore acquire significance beyond clinical best practice because under tort law in many jurisdictions, they are adopted as the benchmark of reasonable professional care against which the conduct of clinicians and laboratories is judged (*154*, *156*).

As NIPT expands to detect more conditions, policy making must consider the societal influences surrounding disability stigmatisation and pregnancy management options such as termination, particularly because the fetus cannot consent or advocate for its rights and the distinction between severe early-onset morbidity and those compatible with a meaningful quality of life can be clinically imprecise (*99*). This complexity is heightened, as expanded NIPT could increasingly inform in utero therapeutics, thus requiring even more robust international legal and regulatory framework (*157*).

Equitable access to NIPT regardless of socioeconomic status, geography, or ethnicity remains far from being met given current cost and technological barriers. Policy frameworks should strengthen the cost-benefit profile of NIPT and thereby increase the likelihood of public funding and equitable access, by both maximizing the clinical value of data already generated by NIPT and expanding the number of conditions detected. Ultimately, realizing the transformative potential of NIPT requires its technical development to proceed in deliberate dialogue with ethical, legal, and regulatory frameworks.

## RESEARCH PRIORITIES

Blood is a complex biofluid, comprising many different soluble and insoluble entities, including EVs and nonvesicular extracellular particles, making it a tremendous analytical challenge to precisely probe the structural topology of cffDNA. While the exact origin and structure of cffDNA in first-trimester maternal blood circulation remain unexpectedly unclear considering the prevalence of NIPT in modern obstetrics, a critical reappraisal of the existing evidence identifies several research priorities.

First, determining the trophoblast cell type origin of cffDNA would likely assist in elucidating the mechanisms involved in its genesis, which could inform the initial structure of cffDNA following release. Second, the involvement of apoptotic processes suggests that, at least at some point, cffDNA is associated with biological extracellular particles. However, the proportion of cffDNA in the maternal blood circulation that remains associated with either vesicular or nonvesicular extracellular particles and how the structural associations differ to cell-free maternal DNA remain to be rigorously determined. This understanding would provide knowledge about a biological characteristic of cffDNA, which may assist in distinguishing fetal and maternal DNA and thereby specifically enable enrichment of cffDNA. Last, it remains also to be determined whether fetal DNA associated with extracellular particles is molecularly identical to total plasma cffDNA used for current NIPT, for example, in fragment size and genomic coverage, and more generally whether it has characteristics suitable for NIPT.

Answering these questions would not only address important knowledge gaps, but it would also likely provide avenues to directly improve NIPT. Knowledge of cffDNA’s cellular origin and structural topology could enable targeted enrichment using techniques such as immunoaffinity enrichment (Fig. 4) (*14*, *158*) or on-chip microfluidic approaches (*159*). These techniques have already been applied to enrich EVs with some success in cancer liquid biopsies (*134*) and could be adapted to enrich other extracellular particles associated with cffDNA. This could, in turn, enable significant increase of the fetal fraction or enrichment of long cffDNA fragments. It therefore stands to reason that a deeper understanding of cffDNA origin and structural topology, combined with new sequencing and artificial intelligence–driven bioinformatic approaches, holds the potential to transform NIPT into a more reliable, comprehensive, and clinically actionable tool with far-reaching benefit in obstetrics. The pace of such technical progress must be met with equally rigorous development of legal and regulatory frameworks, ensuring that as the boundaries of prenatal testing expand, the welfare of both mother and fetus remains the central and nonnegotiable purpose of innovation.
