# Supplementary material for: Reappraising the topology of cell-free fetal DNA in maternal blood toward improved prenatal genetic diagnostics
Source: Sci Adv. 2026 Oct 2;12(40):eaef5425. doi: 10.1126/sciadv.aef5425 (PMC13632725; doi:10.1126/sciadv.aef5425)
Supplement: Supplementary file 1 [file sciadv.aef5425_sm.pdf]

Science Advances
AAAS

Supplementary Materials for

Reappraising the topology of cell-free fetal DNA in maternal blood toward improved prenatal genetic diagnostics

Kieran Sparkes *et al.*

Corresponding author: Marnie Winter, marnie.winter@adelaide.edu.au;
Benjamin Thierry, benjamin.thierry@adelaide.edu.au

Sci. Adv. **12**, eaef5425 (2026)
DOI: 10.1126/sciadv.aef5425

This PDF file includes:

Table S1

Supplementary material:

Table S1. Studies on the association of DNA with extracellular vesicles (EVs) during pregnancy

**Participants**	**Whole blood processing**	**Extracellular Vesicle (EV) isolation method**	**DNA quantification method**	**Findings**	**Refer-ence**
		**DNA enrichment method**	**EV Characterisation method**		
Unknown gestational week (n=28).	Plasma preparation by centrifugation at 800 × g.	Plasma was labelled with a nucleic acid stain and positively stained non-cellular particles sorted using flow cytometry.	Quantitative polymerase chain reaction (qPCR)	2.8% of DNA in non-sorted specimens was fetal.<br><br>28.0% of DNA in DNA$^+$ sorted specimens was fetal.	(*8*)
			Transmission electron microscopy (TEM)		
First, second and third trimester participants (n=9).<br><br>(Preeclamp-tic pregnancy data excluded here).	Plasma preparation by centrifugation at 800 x g for 10 min then 1600 × g for 10 min to obtain large EV supernatant.	EVs were not specifically isolated following plasma preparation.<br><br>Large EVs (0.2-30 µm) analysed by flow cytometry following labelling of human leukocyte antigen-G (HLA-G) or placental alkaline phosphatase positive (PLAP) with a fluorescent antibody.	Flow cytometry with a PicoGreen DNA dye	16.6% of large plasma EVs were HLA-G$^+$.<br><br>16.3% of large plasma EVs were PLAP$^+$.<br><br>Large PLAP$^+$ EVs had 12.2× higher DNA fluorescent intensity than large HLA-G$^+$ EVs.	(*9*)
		No DNA enrichment performed.	Flow cytometry		
Gestational week 13-19 (n=10).	Serum preparation by leaving blood to clot at room temperature	Differential centrifugation: 20,000 × g for 20 min, 0.2 µm filtration, 110,000	qPCR	Serum had 8.3× higher total DNA than plasma.	(*10*)

	then centrifugation at 1880 × g for 10 min.<br><br>Plasma preparation by centrifugation at 1880 × g for 10 min then 2500 × g for 5 min.	× g for 4 h twice (yields exosome pellet).		7.0% of total serum DNA associated with exosomes.<br><br>4.0% of fetal serum DNA associated with exosomes.<br><br>10.3% of total plasma DNA associated with exosomes.<br><br>8.7% of fetal plasma DNA associated with exosomes.	
		Total plasma or serum DNA extracted using QIAamp DSP Virus Kit.<br><br>Exosome DNA extracted using the DNeasy Blood & Tissue Kit (DNase I treated).	Electron microscopy, Western blot		
Gestational weeks 13-27 (n=20).	Plasma preparation by centrifugation at 1600 × g for 10 min then 16,000 × g for 10 min.	ExoQuick™ Exosome Isolation Reagent.	Qubit™ double-stranded (ds) DNA HS Assay (Invitrogen)<br><br>qPCR	44.2% of total plasma DNA associated with EVs (in six samples with detectable EV-associated DNA (EV-DNA)).<br><br>Total DNA 2.8–61.5-fold higher than EV-DNA.<br><br>EV-DNA had a median fragment size of 152.4 base pairs (bp) and 10 bp periodicity.	(*11*)
		Total plasma and exosome DNA extracted using the Hipure Circulating DNA kit (Magen).<br><br>(DNase I treated)	Flow cytometry, TEM, Western blot		
First (n=10), second (n=22) and third trimester (n=49)	Plasma preparation by centrifugation at 300 × g for 10 min then	Invitrogen™ Total Exosome Isolation Kit.	Nanodrop 2000	Average exosome DNA concentration increased 14.1% from trimester 1 to 2 and 45.2% from	(*12*)

participants.	5000 × g for 10 min.	Total plasma DNA extracted using the QIAamp DNA Blood Mini kit.<br><br>Exosome DNA extracted using the Quick-DNA™ Miniprep Plus Kit.	No EV characterisation	trimester two to three.<br><br>52.9% of total plasma DNA associated with exosomes in first trimester, 33.2% in second trimester, and 31.7% in third trimester pregnancies.	
First (n=59), second (n=59), and third trimester (n=59) participants.<br><br>Six participants with an aneuploid fetus.	Plasma preparation by centrifugation at 1600 × g for 10 min then 16,000 × g for 10 min.	Invitrogen™ Total Exosome Isolation (from plasma) kit.	qPCR	The proportion of shorter fetal DNA fragments compared to longer fragments associated with EVs increased with gestation.<br><br>Higher fragmentation of maternal EV-DNA correlated with higher maternal body mass index and age.<br><br>Higher fragmentation of fetal EV-DNA correlated with an aneuploid fetus.	(*13*)
		EV-DNA extracted using the Serum/Plasma Circulating DNA Kit (TIANGEN).<br><br>(DNase I treated)	TEM		
First (n=7), second (n=7), and third trimester (n=7)	Serum preparation by leaving blood to clot for 30 min at room temperature followed by	PLAP^{+} EVs isolated with anti-PLAP antibodies couple with Dynabeads™. EVs isolated using the ExoQuick precipitation	Bisulfite pyrosequencing	50.0% of both EV-DNA and PLAP^{+} EV-DNA was methylated and therefore presumed fetal.	(*14*)

participants.	centrifugation at 2000 × g for 15 min.	solution (System Biosciences).			
		EV-DNA extracted using the Genomic DNA Extraction Kit (GeneAll Biotechnology).	Nanoparticle tracking analysis (NTA), TEM, Western blot		
